# Native collagen hydrogel nanofibres with anisotropic structure using core-shell electrospinning

**DOI:** 10.1038/s41598-018-24700-9

**Published:** 2018-04-19

**Authors:** Yuka Wakuda, Shohei Nishimoto, Shin-ichiro Suye, Satoshi Fujita

**Affiliations:** 1 Department of Frontier Fibre Technology and Science, Graduate School of Engineering, University of Fukui, Fukui, 910-8507 Japan; 2 Life Science Innovation Center, University of Fukui, Fukui, 910-8507 Japan

## Abstract

Collagen hydrogel is a popular extracellular matrix (ECM) material in regenerative medicine and has an isotropic structure. In contrast, native ECM has an anisotropic structure. Electrospinning of collagen dissolved in organic solvents is widely used for fabricating anisotropic collagen nanofibres; however, such fibres are water-soluble and require cross-linking before use as scaffolds for cell culture. Herein, electrospinning using a core-shell nozzle was employed to spin an aqueous acidic solution of collagen and encapsulate it within a shell of polyvinylpyrrolidone (PVP). Subsequently, the core collagen was gelled, and the shell PVP was washed away using a basic ethanol solution to yield anisotropic collagen hydrogel nanofibres. Immunostaining and Fourier transform infrared spectroscopy revealed that the obtained fibres were composed of collagen, and surface PVP was removed completely. Circular dichroism measurements confirmed that the fibres exhibited the triple helical structure characteristic of collagen. Human umbilical vein endothelial cells cultured on the collagen hydrogel fibres were oriented along the fibre direction. Hence, this method is suitable for fabricating fibrous anisotropic collagen hydrogels without chemical and thermal cross-linking, and can facilitate the development of safe medical materials with anisotropy similar to that of native ECM.

## Introduction

Collagen, a primary component of the extracellular matrix (ECM), exists ubiquitously in tissues such as skin, blood vessels, and tendons. It consists of hierarchically ordered fibrils, which are composed of rigid triple helices of three molecular chains^1–3^. This structure provides an ECM with high strength as well as anisotropic mechanical properties^4–6^. Collagen extracted from living tissues is used widely in the form of a hydrogel as a cell scaffold in regenerative medicine to ensure high biocompatibility and bioactivity^7,8^. However, this collagen hydrogel has an isotropic and homogeneous structure, which is different from the anisotropic three-dimensional structure of native living tissues^1,2,9^. In recent years, it has been determined that the geometry and shape of cells and their surrounding environment affect cell behaviour and function^10–14^. Thus, a collagen hydrogel with an anisotropic fibrous structure that mimics the microenvironment of ECM would be highly desirable for use as a cell scaffold, as it would allow for the control of the cellular microenvironment for constructing three-dimensional tissues and organs from cells^9,15–19^. Several methods for obtaining anisotropic collagen hydrogels have been reported. For example, an anisotropic collagen hydrogel can be produced using low interstitial flow^20^, by applying a shear stress with aspiration and injection through a needle^21^, and by using shearing rotary between discs^22^. The repeated lyophilisation of a collagen solution in a columnar tube and the resulting anisotropic shrinkage of the obtained gel yields an anisotropic collagen hydrogel^23^. However, the methods mentioned above are restricted in terms of the shape and size of the gels produced, and more practical and facilitative methods are desired.

Electrospinning is a facile method for obtaining anisotropic nanofibre bundles. In this method, a high voltage is applied to a polymer solution, which is then injected through a needle towards a grounded collector. During this process the solvent evaporates, and nanofibres are obtained^24–27^. For the electrospinning of collagen, the combination with polymers including polylactide^28,29^, polyglycolide^30^, polycaprolactone^31^, polyethylene oxide^32^, and their copolymers^30,33^, in blends or in core-shell was reported, but it has been difficult to obtain pure collagen nanofibres^34^. An aprotic, highly polar organic solvent such as hexafluoroisopropanol (HFIP) or 2,2,2-trifluoroethanol (TFE) must be used to obtain a highly viscous collagen solution^34–36^, because it is known that the viscosity of the polymer solution is a determining factor during the electrospinning process^37,38^. However, these solvents interfere with the hydrogen bonds between the collagen molecules and destroy the ordered triple helical structure^34^. Therefore, collagen fibres obtained by electrospinning are denatured into gelatin fibres, which dissolve in water if not treated with chemical cross-linkers^39^. To the best of our knowledge, no practical method has been reported for fabricating collagen nanofibres by electrospinning while maintaining the higher order structure of native collagen without using a cross-linker.

Herein, we propose a method for electrospinning collagen hydrogel nanofibres using an aqueous collagen solvent while maintaining the triple helical structure of collagen. As mentioned above, the successful electrospinning of an aqueous solution of collagen has not yet been reported. We fabricated core-shell nanofibres in which an aqueous collagen solution formed the nanofibre core within a shell of a water-soluble and spinnable polymer, namely, polyvinylpyrrolidone (PVP). Subsequently, the collagen in the core was subjected to gelation through incubation in a basic solution, and the PVP shell was removed by washing with water to obtain collagen hydrogel fibres without having to use chemical or thermal cross-linking. Further, aligned fibres could be obtained by rotating the collector in a single direction at a high speed.

This is the first study to report that collagen hydrogel nanofibres insoluble in water can be formed by the electrospinning method without using any cross-linkers. Anisotropic collagen hydrogel fibres composed of native collagen fibres exhibiting the triple helical structure can be used as a cell scaffold material with physiological activity similar to that of natural collagen. Further, collagen hydrogel fibres can be bundled to construct tissue-mimicking materials with an anisotropic higher-order structure, such as artificial blood vessels, for medical applications.

## Results

### Electrospinning of collagen/PVP nanofibres

**Figure 1:**
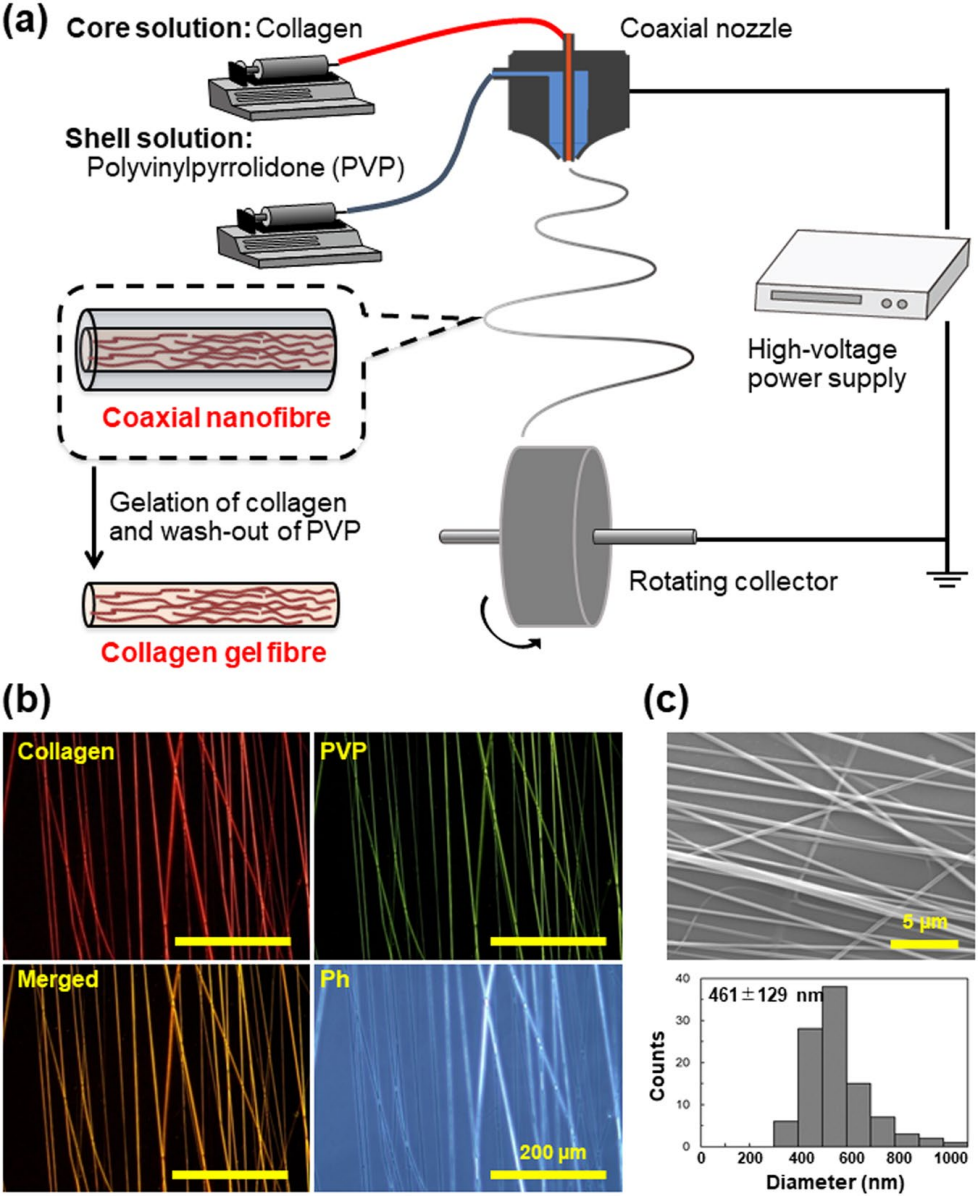
Fabrication of collagen hydrogel nanofibres. (**a**) Schematic illustration of electrospinning and gelation processes. Electrospinning setup consisted of coaxial nozzle and rotating collector. Aqueous collagen and PVP solutions were injected into core-flow and shell-flow chambers, respectively, of the coaxial nozzle. Next, polymer solutions were sprayed under high voltage onto the collector to obtain fibres. Obtained collagen/PVP core-shell nanofibres were gelated and PVP was washed to obtain pure collagen hydrogel nanofibres. (**b**) Morphological analysis of electrospun coaxial fibres. Rhodamine B (red) and uranine (green) were added to 1% collagen (core) solution and 40% PVP (shell) solution, respectively. Bar = 200 µm. (**c**) SEM image and distribution of diameters of electrospun fibres. Bar = 5 µm. Average diameter was 461 ± 129 nm (*n* = 100).

Oriented core-shell-type collagen/PVP nanofibres consisting of a collagen core and a PVP shell were prepared by electrospinning using a coaxial nozzle and a rotating collector (Fig. 1a). First, the concentrations of the polymer solutions used for the electrospinning process were optimised. The concentration of the collagen solution was kept constant at 1%, which is the upper limit, as the solution viscosity should be such that it can be injected. At the same time, the concentration of the PVP solution was varied at 7.5%, 25%, and 40%. The results are shown in Fig. S1. Rhodamine B was added to the collagen solution prior to electrospinning to allow for fluorescence-based observations. When the concentration of the PVP solution was low, both solutions formed beads when electrosprayed. On the other hand, when the PVP solution concentration was higher than 40%, fibrillisation occurred readily. However, for concentrations higher than 40%, it was difficult to handle and inject the PVP solution owing to the higher viscosity. Next, the effect of the flow rate was examined. The flow rate was varied from 0.1 to 1.0 mL·h^−1^. The concentrations of the collagen and PVP solutions were fixed at 1% and 40%, respectively. As shown in Fig. S2, when the flow rate of either the core solution or the shell solution was too high, the eletcrospraying of the solution resulted in the formation of droplets, with no fibres being formed. On the other hand, when the flow rate was too low, continuous fibres were not formed. The optimal concentrations and flow rates of the collagen and PVP solutions were determined to be 1% and 40% and 0.4 and 0.6 mL·h^−1^, respectively. Fibres fabricated under the optimised conditions are shown in Fig. 1b. Rhodamine B (red) and uranine (green) were added to the collagen and PVP solutions, respectively, to allow for fluorescence observations. It was confirmed that both collagen and PVP were colocalised in the nanofibres and that the collagen had been fibrillated without fragmentation. The orientation of the fibres was evaluated quantitatively based on the second-order parameter, *S*, with an *S* value of 1, meaning perfectly aligned fibres, and an *S* value of 0, meaning randomly oriented fibres. The value of the second-order parameter for the electrospun fibres were 0.81 ± 0.03 (Fig. S3a). Scanning electron microscopy (SEM) observations show that uniform fibres with a diameter of 461 ± 129 nm were obtained (Figs 1b and S3b).

### Optimisation of preparation of collagen gel nanofibres

**Figure 2:**
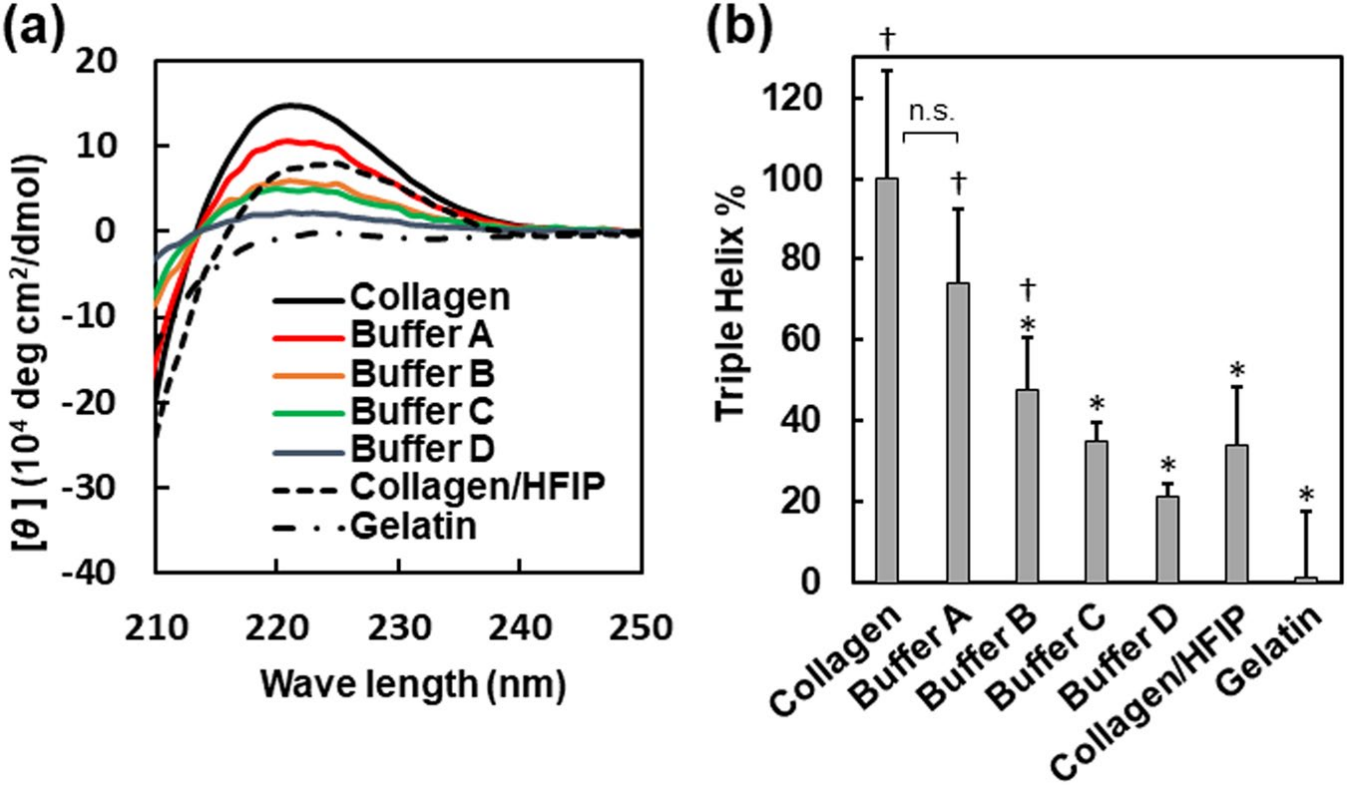
Optimisation of gelation and washing processes. (**a**) Circular dichroism (CD) analysis of gel fibres obtained by washing of electrospun coaxial fibres formed using ethanol solutions of different concentrations. Native collagen hydrogel and gelatin hydrogel were used as positive and negative controls, respectively. Collagen/HFIP nanofibre was also used for comparison. Samples were dissolved in 15 mM acetate buffer for measurement. (**b**) Degree to which triple helical structure was maintained calculated from ${\theta }_{220}$ value (average ± standard deviation, *n* = 3). An asterisk and a dagger represent the significant difference with positive control (native collagen hydrogel) and negative control (gelatin), respectively; ‘n.s.’ represents no significant difference (*p* < 0.05).

To obtain pure collagen hydrogel nanofibres, the electrospun collagen/PVP nanofibres were treated with a basic aqueous solution containing ethanol. This process was performed to form a collagen gel within the fibre core and to remove the PVP shell. Four kinds of basic buffers containing ethanol (Buffers A-D) were tested, pH of which were 8.4, 8.7, 9.6, and 10.3, respectively. The sample obtained after the treatment with the buffer was dissolved and subjected to circular dichroism (CD) measurements, in order to examine whether the higher-order structure of collagen was retained. As can be seen from the CD spectrum, the triple helical structure of collagen exhibits the positive Cotton effect at 220 nm (Fig. 2a). The original collagen, the gelatin, and the collagen fibres electrospun by using single-layer nozzle with HFIP (collagen/HFIP) were used as controls and examined. The residual ratio of the triple helical structure was calculated in terms of the ratio of the molar ellipticity at 220 nm of the sample collagen solution used to that of the original collagen solution. As shown in Fig. 2b, the triple helical structure is barely observed in the case of the gelatin solution. Further, when the fibres were treated with Buffers B, C and D (basic aqueous solutions containing 20%, 50%, and 70% ethanol, respectively), the residual ratios of the triple helical structure were 48%, 35%, and 21%, respectively. However, when Buffer A (PBS containing 20% ethanol) was used, the residual ratio remained at 74%. Therefore, the rest of the experiments were conducted under these conditions.

### Characterisation of collagen hydrogel fibres

#### Fibre morphology

**Figure 3:**
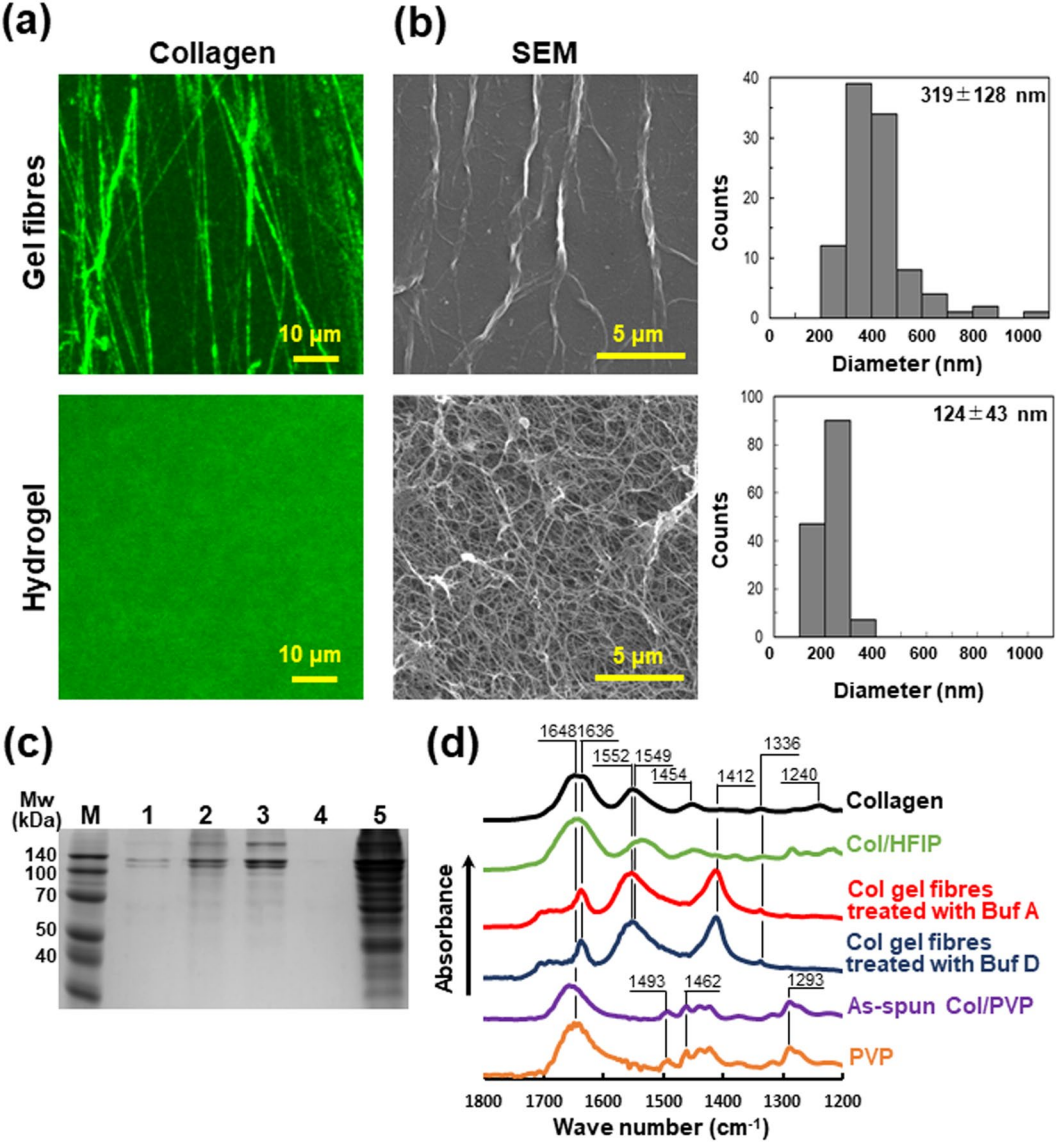
Characterisation of collagen hydrogel nanofibres. (**a,b**) Morphological analysis of collagen hydrogel nanofibres treated with Buffer A (upper) and collagen type I hydrogel (Cellmatrix^®^) as control (lower). (**a**) Fluorescence observations. Samples were stained with anti-collagen type I antibody (green) and captured with a confocal microscope. Negative controls were shown in Fig. S4. (**b**) SEM observations and distribution of fibre diameters of lyophilised samples. Diameter of collagen hydrogel fibres and hydrogel (Cellmatrix^®^) were 319 ± 128 nm (*n* = 101) and 124 ± 43 nm (*n* = 144), respectively. (**c**) SDS-PAGE. Lane 1: as-spun collagen/PVP nanofibres; 2: collagen hydrogel nanofibres washed with Buffer A; 3: native collagen; 4: PVP; and 5: collagen/HFIP nanofibres; M: markers. The electrophoresis was carried out in the same gel. (**d**) ATR-FTIR analysis. Spectra of original collagen (black), collagen/HFIP nanofibres (green), lyophilised collagen hydrogel nanofibres after gelation with Buffer A (red) and Buffer D (blue), collagen/PVP nanofibres before washing (purple), and PVP (orange).

The morphology of the collagen hydrogel fibres obtained from the collagen/PVP nanofibres is shown in Fig. 3a,b. The obtained collagen fibres were stained with an anti-collagen antibody and observed in a confocal microscope. Negative controls were shown in Fig. S4. Clearly, the collagen is exposed to the surface of the fibres and PVP is removed, as the residual PVP on the fibre surface would have inhibited interaction with the anti-collagen antibody. In addition, the obtained fibres were oriented along one direction (*S* = 0.17 ± 0.09, Fig. S3a). Indeed, the anisotropy was lower than before washing, which is due to swelling and loosening during gelation, but the anisotropy achieved during spinning was maintained significantly, compared with the collagen hydrogel which was found to be uniform and isotropic (*S* = 0.02 ± 0.01, Fig. S3a). In the fluorescent observation, the architecture of each fibre in the hydrogel was not observed due to the resolution of the microscope. SEM images show that the diameter of electrospun gel fibres was 319 ± 128 nm (Fig. 3b) and decreased after the treatment with the basic buffer containing ethanol. It was higher than the collagen fibrils of conventional collagen hydrogel (124 ± 43 nm, Figs 3b and S3b). This result implies that some of collagen fibrils became aggregated into individual collagen gel fibre. We also showed that the hydrogel fibres were stably maintained in PBS at 37 °C for 3 days (Fig. S5).

#### SDS-PAGE

The untreated and treated fibres were dissolved in an acetate buffer (pH 3.6) and subjected to sodium dodecyl sulfate polyacrylamide gel electrophoresis (SDS-PAGE). The collagen molecule is composed of two polypeptides, namely, α_1_ and α_2_ chains with molecular weights of 110 kDa and 130 kDa, respectively, and their dimers and trimers are called β and γ chains, respectively^40–42^. As shown in Fig. 3c, three distinct bands related to the α_1_, α_2_, and β chains are observed in the case of the original collagen sample (Lane 3). The collagen/PVP nanofibres and collagen hydrogel fibres obtained after treatment with Buffer A also show the same bands (Lanes 1 and 2). For comparison, conventional electrospinning was performed using a collagen/HFIP solution and a single-layer nozzle, and the fabricated nanofibres were also dissolved and electrophoresed. Interestingly, the bands of the main chains were cleaved into smeared bands, indicating the fragmentation of the peptides (Lane 5).

#### ATR-FTIR

The chemical composition of the obtained hydrogel nanofibres was analysed by attenuated total reflection Fourier transform infrared spectroscopy (ATR-FTIR) (Fig. 3d). The attributions of the various peaks are listed in Table S1. Spectrum of PVP showed characteristic peaks in 1462 cm^−1^ (CH_2_ scissoring) and 1293 cm^−1^ (CN stretching and CH_2_ wagging)^43^; and collagen also showed specific peaks in 1552 cm^−1^ (amide II; CN stretching and NH bending)^44^. In the spectrum of the unwashed collagen/PVP nanofibres, strong peaks attributed to PVP were observed. However, no peaks attributed to collagen were observed. In the spectrum of the hydrogel nanofibres treated with Buffer A or Buffer D, the peak attributable to C-N stretching and CH_2_ wagging was not observed at approximately 1300 cm^−1^. From this result, it can be confirmed that PVP was almost completely removed by the washing process. Finally, the peak at 1412 nm could be attributed to sodium acetate, which was used as a solvent^45^.

### Evaluation of physiological activity on collagen gel fibres by cell culture

**Figure 4:**
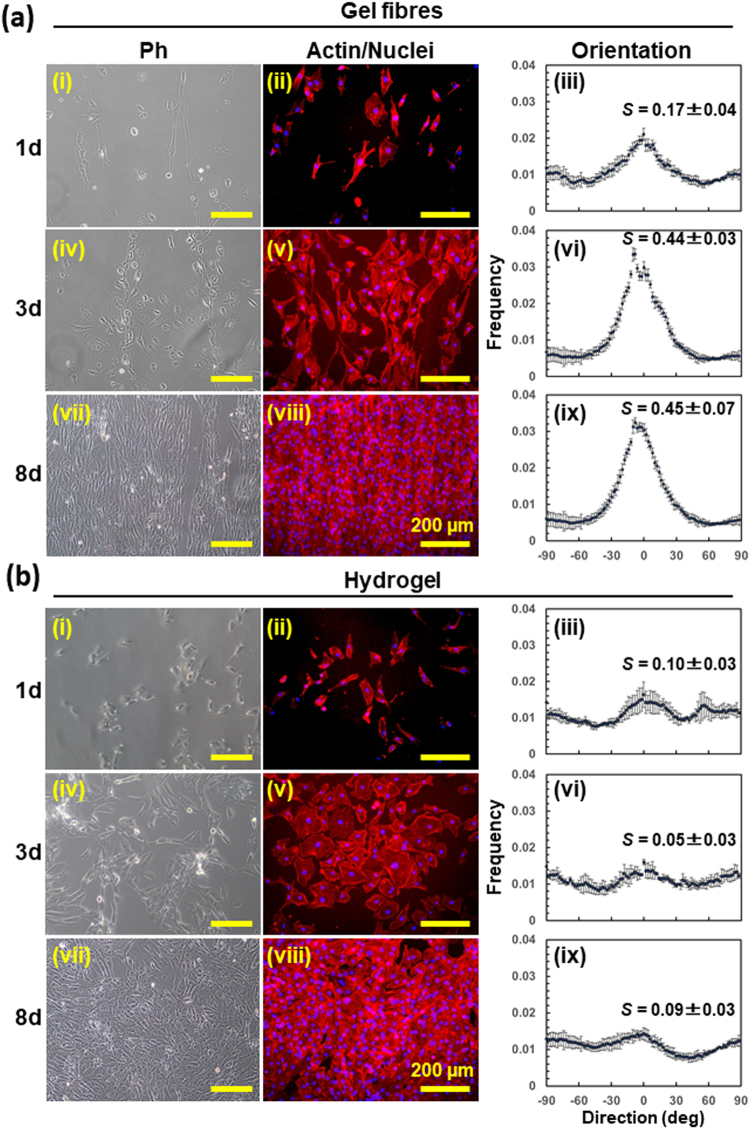
Culture of human umbilical vein endothelial cells (HUVECs) on collagen hydrogel. (**a**) Culture of HUVECs on collagen hydrogel nanofibres. (**b**) Culture on conventional collagen hydrogel deposited on glass slip. Cells were cultured for 1 day (i–iii), 3 days (iv–vi), and 8 days (vii–ix). Immunofluorescence images of HUVECs stained with nuclei (Hoechst, blue) and F-actin (Phalloidin, red). Distributions of cell orientation were calculated based on the averaged power spectra of phase contrast images. Second order parameters, *S*, were represented as the means ± standard deviation (*n* = 3).

Human umbilical vein endothelial cells (HUVECs) were seeded on the fabricated collagen gel fibres, and cell extension was observed. The observations were carried out at day 1, 3, and 8. The results are shown in Fig. 4. The value of the second-order parameter, an index of orientation, for the cells cultured on the hydrogel fibres after 1, 3, and 8 days were 0.17 ± 0.04, 0.44 ± 0.03, and 0.45 ± 0.07, respectively (Fig. S6). On the other hand, the S values of cells cultured on an isotropic hydrogel were 0.10 ± 0.03, 0.05 ± 0.03, and 0.09 ± 0.03 (Fig. S6). From these results, it can be concluded that the cells cultured on the collagen gel fibres extended along the direction of the fibre, whereas those cultured on the ordinary collagen hydrogel did not exhibit anisotropy. In addition, the anisotropy of cell extension on the gel fibres gradually increased with the days of culture. This spontaneous progression of cell anisotropy suggests that some extent of the anisotropy of the gel fibres is sufficient but high anisotropy is not required for the trigger of cell orientation at the beginning of the culture.

## Discussion

Collagen is used widely as a cell scaffold material in biomedical applications in the form of a hydrogel of atelocollagen, which is collagen partially hydrolysed by an acid treatment^1,3,7^. It is soluble in aqueous acidic solutions but maintains its triple helical structure^1,2,6,40^. Further, it forms a homogeneous and isotropic hydrogel through hydrophobic interactions in neutral or basic solutions and a temperature of 37 °C^1,2^. To mimic the anisotropic morphology of the ECM of tissues, it is essential to fabricate collagen hydrogels in the form of fine fibres^1,2,5,7–9^. It has been reported that electrospinning can be used to obtain collagen nanofibres^34–36,39^. However, the electrospinning process destroys the triple helical structure of natural collagen, as it involves the use of highly polar organic solvents, such as HFIP and TFE^36^. As a result, the obtained nanofibres are soluble in water if not treated with a cross-linker, such as glutaraldehyde or carbodiimide. The method proposed in this study is the first one that allows for the fabrication of collagen nanofibre hydrogels by electrospinning in the absence of a cross-linker such that the triple helical structure is maintained. In particular, the molecular chain is fragmented by the electrospinning of the collagen/HFIP solution, as shown in Fig. 3c, because the denatured molecules dissolve in HFIP and get fragmented by the shear stress generated during the spinning process. The thus-obtained collagen hydrogel nanofibres exhibited nondegraded bands during SDS-PAGE, which meant that they probably had the similar higher structure to that of native collagen.

A basic buffer containing ethanol was used for the gelation of the collagen and the removal of the PVP. The CD spectrum of the fibres obtained after the treatment with the basic ethanol solution confirmed that the triple helical structure of collagen was maintained after the washing process (Fig. 2). This result was consistent with those of previous studies. It has been reported that treatment with a low-concentration ethanol solution stabilises the triple helical structure by removing the water hydrated on the protein. On the other hand, treatment with a high-concentration ethanol solution destabilises the collagen structure owing to the hydrophobic interactions with ethanol^40,46^. It has also been reported that it is likely that ethanol enhances the compactness of the polyproline II helices in the triple helical structure of collagen^46,47^.

Furthermore, collagen fibres were observed by immunostaining (Fig. 3a). While peaks related to collagen were detected by FTIR measurements, those related to PVP were not (Fig. 3d). These results suggest that the PVP localised on the fibre surface was completely removed and that the collagen had undergone gelation successfully without being dissolved in water. Indeed, the penetration depth during the ATR-FTIR measurements was approximately 1 μm. However, it is possible that the signal from the nanofibre shell was enhanced as compared to that of the core owing to incomplete optical contact between the ATR crystal and the nanofibre sheet, which had a porous structure^48,49^. During the fluorescence microscopy measurements, the core-shell structure could not be observed directly owing to the resolution limitations related to the observation wavelength. The results of the ATR-FTIR measurements strongly suggested that the collagen/PVP nanofibres had a core-shell double-layered structure, wherein the collagen was covered by PVP.

After the washing process, it is possible that some of the PVP remained within the fibres, although no PVP peaks were detected in ATR-FTIR analysis. However, the trace amount of PVP remaining can be ignored when using the fibres as scaffolds because PVP has low cytotoxicity and has been approved for use in medical applications. For example, it has been reported that when synovial cells derived from rheumatoid arthritis patients were cultured on a material composed of a collagen-PVP composite, they exhibited suppressed expression of the inflammatory cytokines IL-1β and TNF-α^50^. It has also been reported that chondrocytes and synovial cells from osteoarthritis patients, when cultured on collagen-PVP composites, showed proliferation and the suppression of IL-1β and TNF-α^51^.

By using the fabricated anisotropic collagen hydrogel nanofibres, we could successfully control the anisotropy of HUVECs. The electrospinning method allows for the fabrication of a range of anisotropic forms such as tubules, bulk samples, and sheets, which can lead to control the mechanical property of the scaffold. For the potential application to soft tissues, the tuneable elastic modulus of the scaffold is required from 1 kPa to 100 kPa responding to a variety of tissues^52,53^. It is expected that this method will be useful for constructing tissue-like structures. With respect to regenerative medicine, biomaterials that allow for the control of the microenvironment around the cells and the construction of three-dimensional structures for use as tissues and organs are highly desirable^7–9,15–19^. The ECM in the body not only acts as a cell adhesion scaffold but also affects cellular functions, such as proliferation and differentiation, by controlling cell alignment and morphology^7–15^. Thus, for the development of effective biomaterials, one must be able to mimic the physiological activity and anisotropic structure of the ECM. Hence, while anisotropic collagen gels are hard to fabricate, they are essential for regenerative medicine^20–23^. Further, when using collagen nanofibres in medical applications, it is necessary to employ halogen-free solvents and remove any unreacted cross-linkers containing reactive functional groups. The proposed method meets these requirements and has a distinct advantage from a safety viewpoint.

## Methods

### Materials

The following materials were obtained from commercial sources: lyophilised collagen type I from porcine skin (beMatrix^®^ Collagen FD) and collagen type I solution (Cellmatrix^®^) from Nitta Gelatin (Osaka, Japan); lyophilised gelatin (16631-05) from Nacalai Tesque (Kyoto, Japan); PVP (Mw 360,000, K90); Rhodamine B from Wako Pure Chemical Industries (Osaka, Japan); uranine from Tokyo Chemical Industry (Tokyo, Japan); rabbit anti-pig collagen I antibody from Abcam (Cambridge, UK); and Alexa 488-goat anti-rabbit IgG and Alexa 594-Phalloidin from Thermo Fisher Scientific (MA, USA); Hoechst 33342, Dojindo (Kumamoto, Japan). The HUVECs and CS-C medium with CultureBoost-R^™^ were purchased from DS Pharma Biomedical (Osaka, Japan). All other chemicals and reagents used were of analytical grade and were used without further purification.

### Preparation of collagen hydrogel nanofibres

As shown in Fig. 1a, collagen hydrogel nanofibres were obtained using an electrospinning setup consisting of a coaxial nozzle and a rotating collector. The electrospinning solutions were prepared as follows: lyophilised collagen and PVP were dissolved in 15 mM acetate buffer (pH 3.6) to final concentrations of 1% (w/v) and 40% (w/v), respectively. To allow for fluorescence observations, 0.01% Rhodamine B and 0.01% uranine were added to the collagen and PVP solutions, respectively. Collagen/PVP core-shell nanofibres were electrospun on cover slips or aluminium foil using a commercial electrospinning setup (NANON, MECC, Fukuoka, Japan), which consisted of a coaxial spinneret (MECC; core diameter of 0.2 mm, shell diameter of 0.8 mm), syringe pump, high-voltage power supply, and rotating collector placed in a closed chamber. The conditions for the electrospinning process were as follows: applied electric field of 3.6 kV·cm^−1^; flow rate of 0.4 mL·h^−1^ for the core solution (collagen), and 0.6 mL·h^−1^ for the shell solution (PVP); collector rotation speed of 1,500 rpm (linear velocity of 15 m·s^−1^) for ensuring aligned fibres; and spinning time of 3 h. For the fabrication of conventional collagen nanofibres by using HFIP as a solvent (collagen/HFIP nanofibres), 10% collagen dissolved in HFIP was electrospun with a single-layer nozzle in the conditions as follows: applied electric field of 3.7 kV·cm^−1^; flow rate of 5 mL·h^−1^; collector rotation speed of 600 rpm (linear velocity of 6 m·s^−1^; and spinning time of 20 min.

The collagen/PVP core-shell nanofibre sheet electrospun on aluminium foil was cut into pieces with an area of 1 cm^2^, placed in a 35-mm dish, soaked in 2 mL of the washing solution, treated with basic buffers containing ethanol. The following buffers were used. Buffer A: PBS containing 20% ethanol; final salt concentration of 110 mM NaCl, 47 mM Na_2_HPO_4_, 2.1 mM KCl, and 1.2 mM KH_2_PO_4_; Buffer B, C and D: 3 mM aqueous Na_2_HPO_4_ solution with 20%, 50%, and 70% ethanol, respectively. Subsequently, the solution was exchanged with 2 mL of pure water and incubated for 3 h at 37 °C to remove the PVP shell and obtain collagen hydrogel fibres.

### Scanning electron microscopy (SEM)

Collagen gel samples containing water were fixed with 4% paraformaldehyde for 15 min, dehydrated by sequentially and gently being immersed in serially diluted ethanol solutions (50, 60, 70, 80, 90, 95 and 100%) for 10 min and *t*-butyl alcohol 3 times for 10 min each, and then lyophilised overnight using a freeze dryer (FDU-830, EYELA, Tokyo, Japan). The samples were then sputtered with Pt/Pd using an ion sputter (MSP-1S, Vacuum Device Inc., Ibaragi, Japan) for 120 s and imaged using SEM (JSM-6370A, JEOL, Tokyo, Japan) at an accelerating voltage of 12 kV. The diameter of the fibres was examined at 10 randomly selected areas of the scaffolds using an image processing software (fiji.sc; ImageJ, NIH, USA).

### Circular dichroism (CD) spectrometry

All CD measurements were performed using a spectropolarimeter (J-725, JASCO, Tokyo, Japan) with a 1-mm quartz cell. The samples were dissolved in a 15-mM acetate buffer (pH 3.6) before the measurements. For all the measurements, the spectrum wavelength, temperature, scanning rate, resolution, data pitch, response time, and accumulation were 210–250 nm, 25 °C, 50 nm·min^−1^, 1 nm, 1 s, and 4 times, respectively. The extent of recovery of the triple helical structure of collagen after washing with the different washing solutions was calculated using the ellipticity at 220 nm. The value of the original collagen and gelatin was set as 100% and 0%, respectively.

### Sodium dodecyl sulfate-polyacrylamide gel electrophoresis (SDS-PAGE)

The test sample was dissolved in a concentration of 1 mg·mL^−1^ in 0.47 M Tris-HCl buffer (pH 6.8) containing 10% SDS and 10% 2-mercaptoethanol, boiled for 10 min at 90 °C, and electrophoresed in 10% polyacrylamide gel at 100 V for 2 h. WIDE-VIEW Prestained Protein Size Marker (Wako Pure Chemical, Osaka, Japan) was used as the molecular mass standard. The protein bands were stained with Coomassie brilliant blue using RAPID KANTO CBB (Kanto Chemical, Tokyo, Japan) and visualised using the Gel Doc EZ Imager system (Biorad, CA, USA).

### Attenuated total reflection Fourier transform infrared (ATR-FTIR) spectroscopy

The ATR-FTIR spectra of the nanofibres were measured with a Nicolet 6700 system (Thermo Scientific). The test sample was dissolved in a 15-mM acetic buffer (pH 3.6) and lyophilised. The measurements were performed for wavenumbers of 4000 to 800 cm^−1^ at a resolution of 4 cm^−1^ using a KBr detector. An average of 32 scans were performed.

### Cell culture

The HUVECs were seeded at a density of 5 × 10^4^ cells·cm^−2^ and maintained in CS-C medium, which was supplemented with CultureBoost-R^™^ according to the manufacturer’s instructions, at 37 °C in a humidified atmosphere containing 5% CO_2_. The collagen hydrogel fibres were prepared on a cover slip (18 × 24 mm) as described above. As a control scaffold, a collagen type I hydrogel (Cellmatrix^®^) was prepared on a 35-mm dish as per the manufacturer’s instructions.

### Microscopy

Samples were fixed with 4% paraformaldehyde for 15 min and permeabilised with 0.05% Triton-X (Wako) for 10 min 3 times, washed with Blocking One (Nacalai) twice, incubated with the primary antibody, namely, rabbit anti-pig collagen I antibody (1:200 dilution) overnight at 4 °C, washed with 0.05% polyoxyethylene sorbitan monolaurate (Tween 20, Wako) for 15 min 3 times at room temperature, and finally treated with the secondary antibody, namely, Alexa 488-goat anti-rabbit IgG (1:200 dilution), for 2 h at room temperature. For cell staining, Alexa 594-conjugated Phalloidin (1:200) and Hoechst 33342 (1:2000) were used. The samples were observed using an inverted fluorescence microscope (CKX41, Olympus, Tokyo, Japan) and a confocal laser scanning microscope (FV1200, Olympus).

### Analysis of fibre and cell orientation

The fibre and cell orientation were quantified based on a second-order parameter, which was calculated from the power spectrum of the Fourier transform of the images using an image processing software (ImageJ; plugin: directionality). The second-order parameter in a 2D plane, *S*, was defined as follows:

$$S=2\langle {\cos }^{2}\theta \rangle -1=\langle \cos \,2\theta \rangle $$

where *θ* is the orientation angle and 〈cos2*θ*〉 is the average of cos2*θ*^27,54^.

### Statistical analysis

Tukey-Kramer tests were performed using R (Version 3.3.1). *p* < 0.05 was considered statistically significant.

## Electronic supplementary material

Supplementary Info

## References

[1] Kotch FW, Raines RT (2006). Self-assembly of synthetic collagen triple helices. Proc. Natl. Acad. Sci..

[2] Shoulders MD, Raines RT (2009). Collagen structure and stability. Annu. Rev. Biochem..

[3] Slaughter BV, Khurshid SS, Fisher OZ, Khademhosseini A, Peppas NA (2009). Hydrogels in regenerative medicine. Adv. Mater..

[4] Meyers MA, Chen P-Y, Lopez MI, Seki Y, Lin AYM (2011). Biological materials: a materials science approach. J. Mech. Behav. Biomed. Mater..

[5] Gautieri A, Vesentini S, Redaelli A, Buehler MJ (2011). Hierarchical structure and nanomechanics of collagen microfibrils from the atomistic scale up. Nano Lett..

[6] Ohyabu Y, Yunoki S, Hatayama H, Teranishi Y (2013). Fabrication of high-density collagen fibril matrix gels by renaturation of triple-helix collagen from gelatin. Int. J. Biol. Macromol..

[7] Antoine EE, Vlachos PP, Rylander MN (2014). Review of collagen I hydrogels for bioengineered tissue microenvironments: characterization of mechanics, structure, and transport. Tissue Eng. Part B Rev..

[8] Cheema U, Brown RA (2013). Rapid Fabrication of Living Tissue Models by Collagen Plastic Compression: Understanding Three-Dimensional Cell Matrix Repair *In Vitro*. Adv. Wound Care.

[9] Walters BD, Stegemann JP (2014). Strategies for directing the structure and function of three-dimensional collagen biomaterials across length scales. Acta Biomater..

[10] McBeath R, Pirone DM, Nelson CM, Bhadriraju K, Chen CS (2004). Cell shape, cytoskeletal tension, and RhoA regulate stem cell lineage commitment. Dev. Cell.

[11] Discher DE, Janmey P, Wang YL (2005). Tissue cells feel and respond to the stiffness of their substrate. Science.

[12] Lecuit T, Lenne PF (2007). Cell surface mechanics and the control of cell shape, tissue patterns and morphogenesis. Nat. Rev. Mol. Cell Biol..

[13] Dahl KN, Ribeiro AJS, Lammerding J (2008). Nuclear shape, mechanics, and mechanotransduction. Circ. Res..

[14] Doyle AD, Yamada KM (2016). Mechanosensing via cell-matrix adhesions in 3D microenvironments. Exp. Cell Res..

[15] Tibbitt MW, Anseth KS (2009). Hydrogels as extracellular matrix mimics for 3D cell culture. Biotechnol. Bioeng..

[16] Miron-Mendoza M, Koppaka V, Zhou C, Petroll WM (2013). Techniques for assessing 3-D cell–matrix mechanical interactions *in vitro* and *in vivo*. Exp. Cell Res..

[17] Verhulsel M (2014). A review of microfabrication and hydrogel engineering for micro-organs on chips. Biomaterials.

[18] Ahadian S (2015). Hybrid hydrogels containing vertically aligned carbon nanotubes with anisotropic electrical conductivity for muscle myofiber fabrication. Sci. Rep..

[19] Nguyen LH (2017). Three-dimensional aligned nanofibers-hydrogel scaffold for controlled non-viral drug/gene delivery to direct axon regeneration in spinal cord injury treatment. Sci. Rep..

[20] Ng CP (2005). Interstitial fluid flow induces myofibroblast differentiation and collagen alignment *in vitro*. J. Cell Sci..

[21] Marelli B, Ghezzi CE, James-Bhasin M, Nazhat SN (2015). Fabrication of injectable, cellular, anisotropic collagen tissue equivalents with modular fibrillar densities. Biomaterials.

[22] Yunoki S, Hatayama H, Ebisawa M, Kondo E, Yasuda K (2015). A novel fabrication method to create a thick collagen bundle composed of uniaxially aligned fibrils: an essential technology for the development of artificial tendon/ligament matrices. J. Biomed. Mater. Res. Part A.

[23] Lowe CJ, Reucroft IM, Grota MC, Shreiber DI (2016). Production of Highly Aligned Collagen Scaffolds by Freeze-drying of Self-assembled, Fibrillar Collagen Gels. ACS Biomater. Sci. Eng..

[24] Barnes CP, Sell SA, Boland ED, Simpson DG, Bowlin GL (2007). Nanofiber technology: designing the next generation of tissue engineering scaffolds. Adv. Drug Deliv. Rev..

[25] Bhardwaj N, Kundu SC (2010). Electrospinning: a fascinating fiber fabrication technique. Biotechnol. Adv..

[26] Theron A, Zussman E, Yarin AL (2001). Electrostatic field-assisted alignment of electrospun nanofibres. Nanotechnology.

[27] Batnyam O (2015). Biohybrid hematopoietic niche for expansion of hematopoietic stem/progenitor cells by using geometrically controlled fibrous layers. RSC Adv..

[28] Kim K (2003). Control of degradation rate and hydrophilicity in electrospun non-woven poly(D,L-lactide) nanofiber scaffolds for biomedical applications. Biomaterials.

[29] He W, Yong T, Teo WE, Ma Z, Ramakrishna S (2005). Fabrication and endothelialization of collagen-blended biodegradable polymer nanofibers: Potential vascular graft for blood vessel tissue engineering. Tissue Eng..

[30] Li M (2006). Co-electrospun poly(lactide-co-glycolide), gelatin, and elastin blends for tissue engineering scaffolds. J. Biomed. Mater. Res. Part A.

[31] Schnell E (2007). Guidance of glial cell migration and axonal growth on electrospun nanofibers of poly-ε-caprolactone and a collagen/poly-ε-caprolactone blend. Biomaterials.

[32] Huang L, Nagapudi K, P.Apkarian R, Chaikof EL (2001). Engineered collagen–PEO nanofibers and fabrics. J. Biomater. Sci. Polym. Ed..

[33] Yan S, Xiaoqiang L, Lianjiang T, Chen H, Xiumei M (2009). Poly(l-lactide-co-ɛ-caprolactone) electrospun nanofibers for encapsulating and sustained releasing proteins. Polymer.

[34] Bürck J (2013). Resemblance of electrospun collagen nanofibers to their native structure. Langmuir.

[35] Zhong S (2006). An aligned nanofibrous collagen scaffold by electrospinning and its effects onin vitro fibroblast culture. J. Biomed. Mater. Res. Part A.

[36] Zeugolis DI (2008). Electro-spinning of pure collagen nano-fibres - Just an expensive way to make gelatin?. Biomaterials.

[37] Gupta P, Elkins C, Long TE, Wilkes GL (2005). Electrospinning of linear homopolymers of poly(methyl methacrylate): exploring relationships between fiber formation, viscosity, molecular weight and concentration in a good solvent. Polymer.

[38] Thompson CJ, Chase GG, Yarin AL, Reneker DH (2007). Effects of parameters on nanofiber diameter determined from electrospinning model. Polymer.

[39] Xing Q (2014). Increasing mechanical strength of gelatin hydrogels by divalent metal ion removal. Sci. Rep..

[40] Engel, J. & Bächinger, H. P. Structure, Stability and Folding of the Collagen Triple Helix in *Topics in Current Chemistry* (eds. Brinckmann, J., Notbohm, H. & Müller, P. K.) **98**, 7–33 (Springer, 2005).

[41] Ogawa M (2003). Biochemical properties of black drum and sheepshead seabream skin collagen. J. Agric. Food Chem..

[42] Banerjee I, Mishra D, Das T, Maiti S, Maiti TK (2012). Caprine (goat) collagen: a potential biomaterial for skin tissue engineering. J. Biomater. Sci. Polym. Ed..

[43] Dumitraşcu M (2011). Characterization of electron beam irradiated collagen- polyvinylpyrrolidone (PVP) and collagen-dextran (DEX) blends. Dig. J. Nanomater. Biostructures.

[44] Borodko Y (2006). Probing the interaction of poly(vinylpyrrolidone) with platinum nanocrystals by UV-Raman and FTIR. J. Phys. Chem. B.

[45] Maiorov VD, Burdin VV, Voloshenko GI, Librovich NB (1996). Ions with a strong symmetric H-bond in solutions of sodium acetate in acetic acid. Russ. Chem. Bull..

[46] Jiang Q, Reddy N, Zhang S, Roscioli N, Yang Y (2013). Water-stable electrospun collagen fibers from a non-toxic solvent and crosslinking system. J. Biomed. Mater. Res. Part A.

[47] Gopinath A, Reddy SMM, Madhan B, Shanmguam G, Rao JR (2014). Effect of aqueous ethanol on the triple helical structure of collagen. Eur. Biophys. J..

[48] Park SC, Liang Y, Lee HS (2004). Quantitative Analysis Method for Three-Dimensional Orientation of PTT by Polarized FTIR-ATR Spectroscopy. Macromolecules.

[49] Yang P (2005). Thickness measurement of nanoscale polymer layer on polymer substrates by attenuated total reflection infrared spectroscopy. Anal. Chem..

[50] Furuzawa-Carballeda J, Rodríquez-Calderón R, Díaz de León L, Alcocer-Varela J (2002). Mediators of inflammation are down-regulated while apoptosis is up-regulated in rheumatoid arthritis synovial tissue by polymerized collagen. Clin. Exp. Immunol..

[51] Furuzawa-Carballeda J, Muñoz-Chablé OA, Barrios-Payán J, Hernández-Pando R (2009). Effect of polymerized-type I collagen in knee osteoarthritis. I. *In vitro* study. Eur. J. Clin. Invest..

[52] Zhu Y, Dong Z, Wejinya UC, Jin S, Ye K (2011). Determination of mechanical properties of soft tissue scaffolds by atomic force microscopy nanoindentation. J. Biomech..

[53] Skardal A (2015). A hydrogel bioink toolkit for mimicking native tissue biochemical and mechanical properties in bioprinted tissue constructs. Acta Biomater..

[54] Reffay M (2011). Orientation and Polarity in Collectively Migrating Cell Structures: Statics and Dynamics. Biophys. J..

