# Supplementary material for: Native collagen hydrogel nanofibres with anisotropic structure using core-shell electrospinning
Source: Sci Rep. 2018 Apr 19;8:6248. doi: 10.1038/s41598-018-24700-9 (PMC5908855; doi:10.1038/s41598-018-24700-9)
Supplement: Supplementary file 1 — Supplementary Info [file 41598_2018_24700_MOESM1_ESM.docx]

**Supplementary Information**

**Native collagen hydrogel nanofibres with anisotropic structure using core-shell electrospinning**

Yuka Wakuda^a^, Shohei Nishimoto^a^, Shin-ichiro Suye^a,b^, and Satoshi Fujita^a,b^ *

^a^Department of Frontier Fibre Technology and Science, Graduate School of Engineering, University of Fukui, Fukui, 910-8507, Japan

^b^Life Science Innovation Center, University of Fukui, Fukui, 910-8507, Japan

*Corresponding author at: fujitas@u-fukui.ac.jp

**
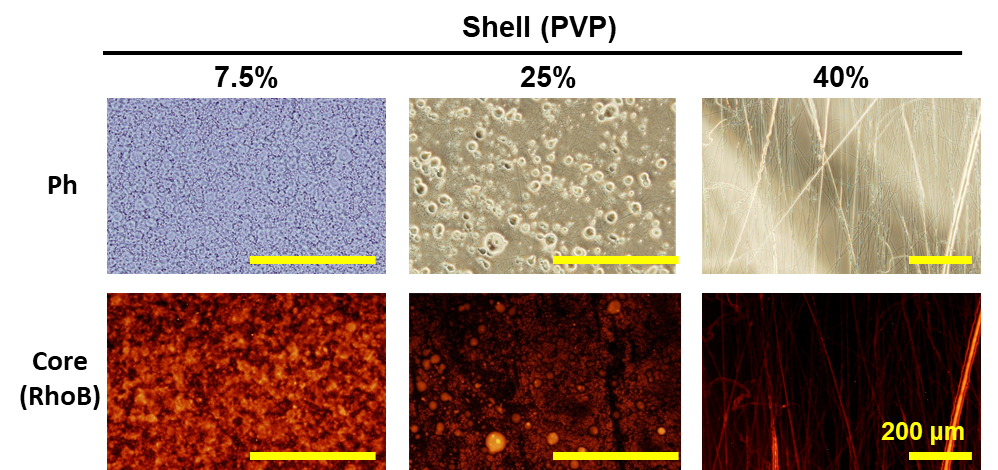
Fig. S1 Optimisation of polymer concentration.** Microscopy images of fibres fabricated from polymer solution and 1.0 w/v% collagen (as core solution and containing Rhodamine B (red)) and PVP solutions of different concentrations (as shell solution). Upper images are phase-contrast images while lower images are fluorescence images. Flow rate, electric field, and collector rotation were 0.6/1.0 mL·h^-1^ (core/shell), 3.7 kV·cm^-1^, and 15 m·s^-1^, respectively. Bars = 200 µm.

**
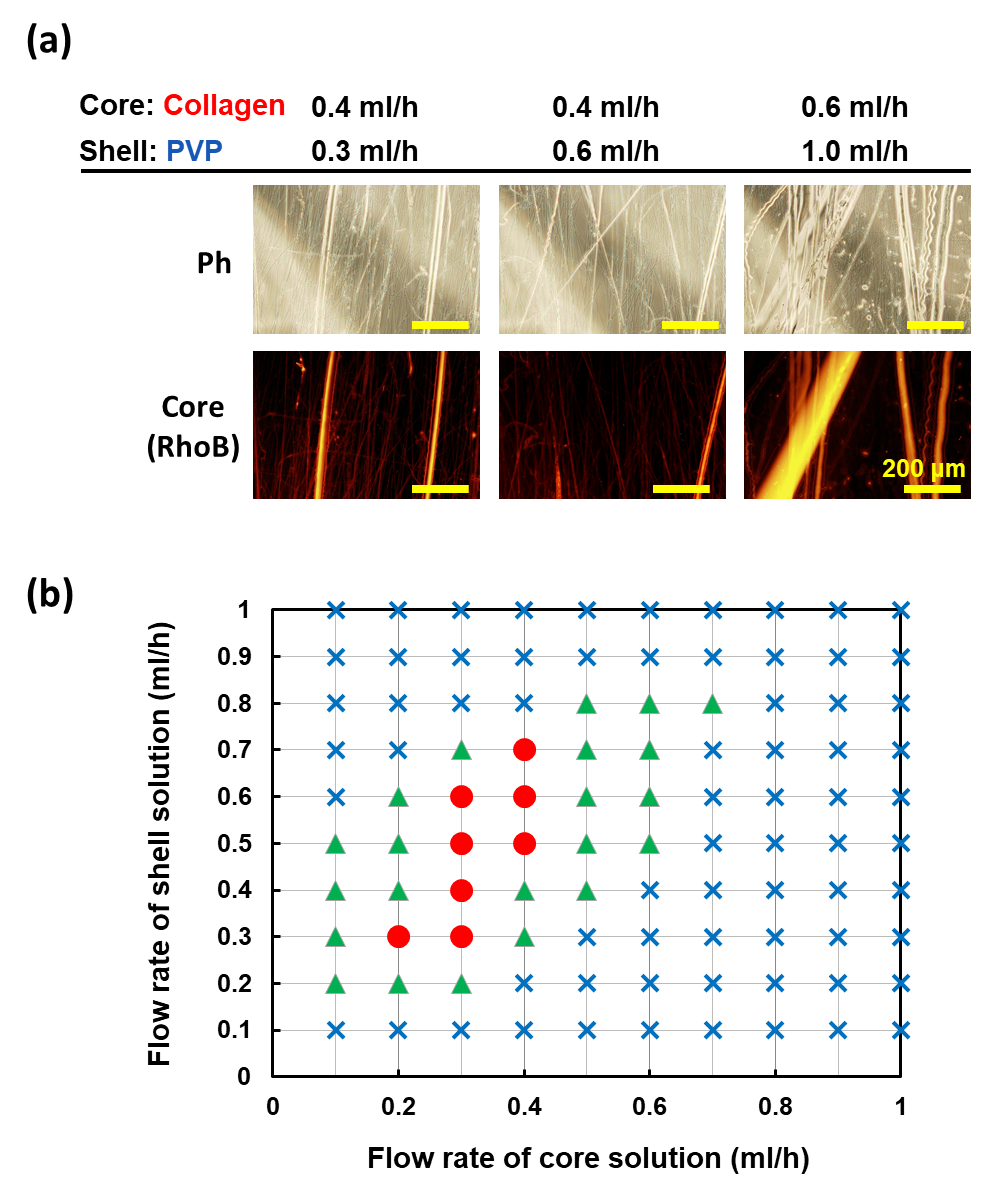
Fig. S2** **Optimisation of flow rates. (a)** Microscopy images of coaxial nanofibres electrospun using different flow rates. Concentrations of collagen and PVP solutions were 1.0 w/v% and 40%, respectively. Collagen solution contained Rhodamine B (red). Upper panels are phase-contrast images while lower panels are fluorescence images. Flow rates, electric field, and collector rotation were 0.4/0.3, 0.4/0.6, and 0.6/1.0 mL·h^-1^ (core/shell); 3.7 kV·cm^-1^, and 15 m·s^-1^, respectively. Bars = 200 µm. **(b)** Degree of fibrillisation at different flow rates. Circles indicate that fine fibres were obtained, triangles indicate that either incomplete fibres or droplets were formed, and crosses indicate that no fibres were formed.

**
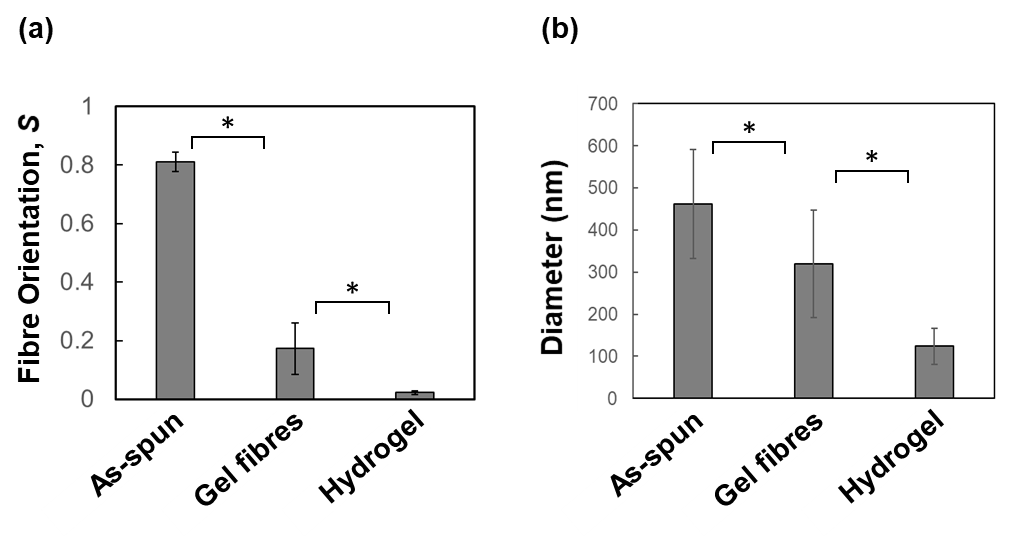
Fig. S3 Statistical comparison of fibre orientation and diameter. (a)** Second order parameters, *S*, were calculated based on the averaged power spectra of fluorescent images (n = 3–5). **(b)** Diameter was measured from SEM images. The values were represented as the means ± standard deviation. An asterisk represents significant difference (*p* < 0.05). As-spun: collagen/PVP nanofibres before washing; Gel fibres: collagen hydrogel nanofibres after gelation with Buffer A; Hydrogel: collagen type I hydrogel (Cellmatrix^®^).

**
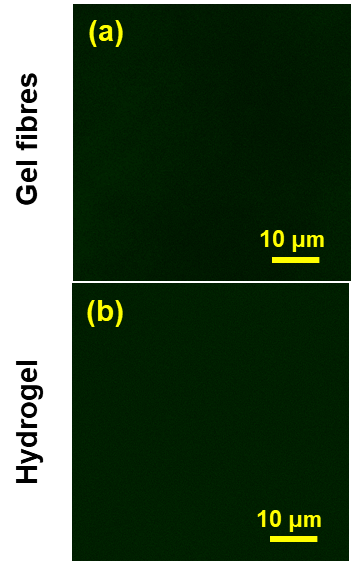
Fig. S4 Negative controls of immunostaining corresponding to Fig. 3a.** Fluorescence images of collagen hydrogel nanofibres treated with Buffer A **(a)** and collagen type I hydrogel (Cellmatrix^®^) as control **(b)**. Samples were stained with only second antibody (green) and captured with a confocal microscope. All contrast parameters were the same as **Fig. 3a**.

**
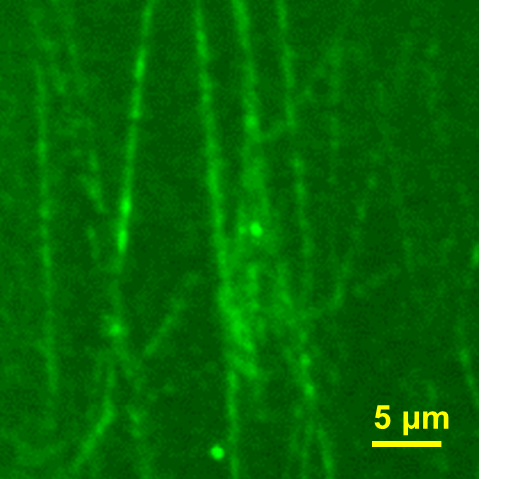
Fig. S5 Long-term stability of collagen hydrogel nanofibres.** Confocal fluorescent image of collagen hydrogel nanofibres after the incubation at 37°C for 3 days. Fibres maintained their fibrous structure and orientation for 3 days.

**
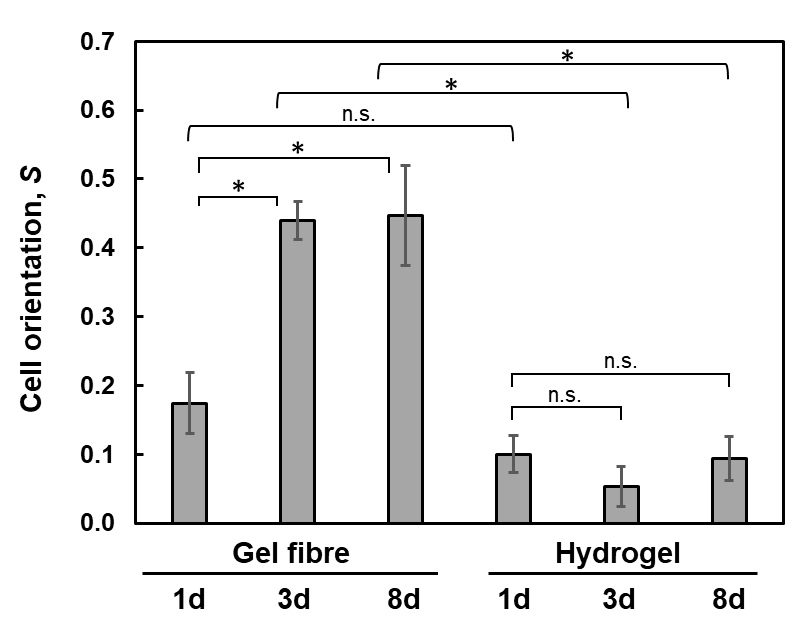
Fig. S6 Statistical comparison of cell orientation.** Second order parameters, *S*, of cell orientation were represented as the means ± standard deviation (*n* = 3). An asterisk and ‘n.s.’ represent significant and no significant difference, respectively.

**Table S1 Attribution of ATR-FTIR peaks.**

| Materials | Peaks (cm^-1^) | Attributions |
| --- | --- | --- |
| Collagen | 1240 | N-H in-plane bending and C-N stretching (Amide III) |
|  | 1336, 1454 | CH_2_ bending, CH_2_ bending |
|  | 1552 | C-N stretching and N-H bending (Amide II) |
|  | 1648 | C=O stretching (Amide I) |
| PVP | 1293 | C-N stretching and CH_2_ wagging |
|  | 1462 | CH_2_ scissoring |
|  | 1493 | C-N scissoring |
|  | 1648 | C=O stretching (Amide I) |
